# ^31^P Dynamic Nuclear Polarization through the Solid Effect: Study of Biomolecules in Aqueous Solutions at 9.4 T

**DOI:** 10.1021/acs.analchem.5c02158

**Published:** 2025-07-09

**Authors:** Andrei Kuzhelev

**Affiliations:** 260754 Goethe University Frankfurt am Main, Institute of Physical and Theoretical Chemistry and Center for Biomolecular Magnetic Resonance, Max von Laue Str. 7, 60438 Frankfurt am Main, Germany

## Abstract

Dynamic Nuclear Polarization (DNP) has emerged as a transformative technique for enhancing the sensitivity of Nuclear Magnetic Resonance (NMR) spectroscopy. In the quest to improve the polarization of ^31^P nuclei in liquid environments, previous efforts have yielded only limited success. This study introduces a novel approach utilizing the solid effect to effectively polarize pentavalent phosphorus within the phosphate groups of key biomolecules, including adenosine triphosphate, nucleic acid, and lipid bilayer in the fluid phase. We report a remarkable ^31^P DNP enhancement factor exceeding 20 for these compounds in aqueous solutions at high magnetic fields and ambient temperatures. These findings not only highlight the potential of solid-effect DNP for liquid systems but also open new avenues for advanced investigations into the dynamics and binding interactions of biomolecules.

## Graphical abstract

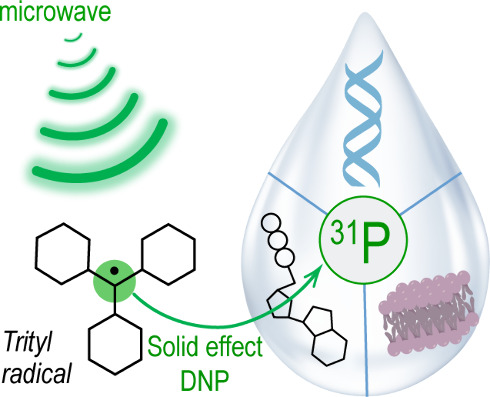

Nuclear Magnetic Resonance (NMR) spectroscopy is a powerful analytical tool widely used to investigate biomolecules, providing deep insights into their structure, dynamics, and interactions. Despite its extensive utility, NMR spectroscopy is often hampered by sensitivity limitations, especially when applied to nuclei with low gyromagnetic ratios, such as ^31^P, ^13^C, and ^15^N, which are essential for probing biological processes. Dynamic Nuclear Polarization (DNP) has emerged as a promising strategy to enhance NMR sensitivity by transferring polarization from electron spins to nuclear spins.^1^

In this study, we direct our focus toward phosphorus-31, a nucleus routinely employed in NMR studies involving membranes,^2^ metabolomics,^3^ and the structural analysis of nucleic acids.^4^ Previous DNP research on ^31^P nuclei in liquid states have utilized dissolution DNP^5–7^ and the Overhauser effect.^8–12^

Dissolution DNP enhances nuclear spin polarization at cryogenic temperatures, enabling the study of liquid-state NMR of highly polarized samples.^13^ This technique has revealed a number of applications for phosphorus-31, including pH imaging of phosphate solutions,^5^ studying calcium phosphate crystal growth,^6^ and monitoring the hydrolysis reaction of pyrophosphates.^7^ Despite ^31^P natural abundance of 100% and its presence in various biomolecules, the application of dissolution DNP is limited by the short lifetime of the hyperpolarized state. This limitation primarily arises from the relatively fast spin–lattice relaxation time (*T*_1_) of ^31^P nuclei, which restricts the full potential of this powerful technique.

The Overhauser DNP relies on the modulation of the electron–nuclear hyperfine interaction on a time scale comparable to the reciprocal electron Larmor frequency.^14–16^ Previous research has demonstrated that the effectiveness of Overhauser DNP is strongly influenced by the chemical environment surrounding the ^31^P nucleus. In sterically accessible trivalent phosphorus, scalar relaxation predominantly drives the enhancement,^9^ whereas dipolar interactions play a more critical role in pentavalent phosphorus compounds.^11,12^ There is one notable example of a successful polarization of the ^31^P nucleus using the Overhauser effect, where an enhancement of >160 has been obtained across magnetic fields ranging from 1.2 to 14.1 T.^17–22^ However, this achievement is restricted to specific combinations involving triphenylphosphine (with trivalent phosphorus) as the analyte and 1,3-bis­(diphenylene)-2-phenylallyl (BDPA) radical as the polarizing agent. These particular components enable a strong Fermi contact interaction between the polarizing agent and the analyte, resulting in a large ^31^P DNP enhancement. On the downside, the Overhauser effect is ineffective for triphenylphosphine oxide,^20^ which contains pentavalent phosphorus, highlighting the limitations of the technique.

Given that ^31^P nuclei in biological molecules exist predominantly in the pentavalent state and exhibit fast *T*_1_, the inefficiency of the Overhauser effect and dissolution DNP for these compounds emphasizes the need for alternative methods. In this manuscript, we propose a novel approach for hyperpolarization of phosphorus-31 in aqueous solutions based on the solid-effect DNP. Our research focuses on key biomolecules such as adenosine triphosphate (ATP), double-stranded DNA, and lipid bilayers in their fluid phase (see Figure 1). We demonstrate a significant ^31^P DNP enhancement factor exceeding 20 for these compounds, thereby improving the sensitivity of NMR at room temperatures and high magnetic fields.

**Figure 1:**
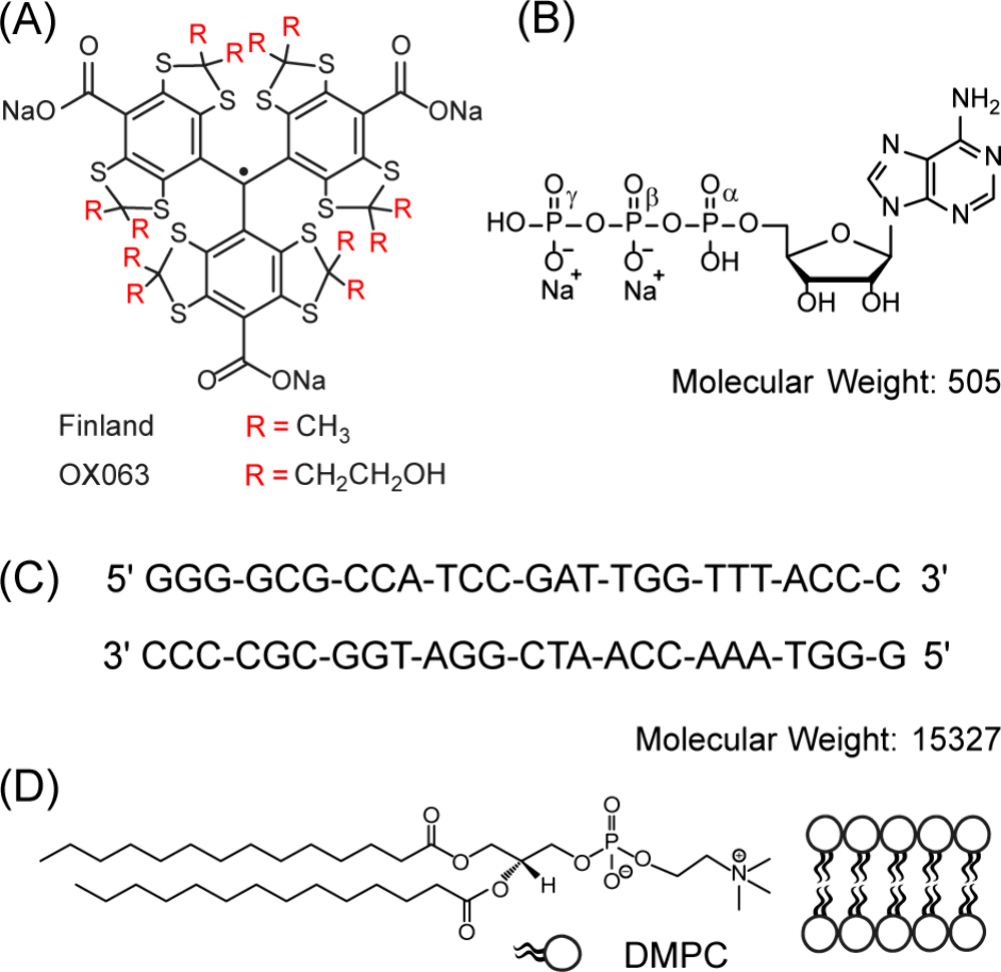
Chemical structure of studied samples: (A) triarylmethyl radicals (Finland and OX063), (B) adenosine triphosphate (ATP), (C) 25-mer DNA duplex, and (D) DMPC lipid bilayers.

The solid effect is a two-spin process characterized by the dipolar interaction between an electron and a nucleus.^23,24^ It occurs when a system, typically a mixture of polarizing agents and analytes, is irradiated at frequencies of ω_e_ ± ω_n_, where ω_e_ and ω_n_ represent the electron and nuclear Larmor frequencies, respectively. This irradiation excites electron–nuclear transitions that are formally forbidden. However, the pseudosecular components of the dipolar coupling cause very weak mixing between the spin states, allowing for these transitions to become partially permitted. This effect is particularly relevant in environments with restricted molecular motion, such as the solid state. Additionally, its effectiveness is inversely proportional to the square of the magnetic field strength, necessitating a strong microwave magnetic field (*B*_1_). Recent studies have highlighted the potential of the solid effect in enhancing nuclear polarization, especially with narrow-line polarizing agents.^25–28^ Notably, a significant ^1^H DNP enhancement has been achieved in *ortho*-terphenyl at a magnetic field of 9.4 T and a temperature of 100 K, with enhancement factor exceeding 500 (∼80% of the theoretical maximum, γ_e_/γ_H_ ≈ 660), when sufficient microwave field strength (*B*_1_) is applied.^29^

For many years, the prevailing belief was that the solid effect was effective only in solid-state conditions, particularly at high magnetic fields. However, recent research has broadened this perspective, demonstrating its potential for hyperpolarizing nuclei in viscous liquids and heterogeneous systems.^30–32^ In solid-state conditions, spin diffusion plays a significant role in polarization transfer; conversely, in liquid states, polarization is directly transferred from the electron spin of the polarizing agent to the nearest nuclei of the analyte. Subsequent propagation of polarization to other molecules occurs through molecular diffusion. Triarylmethyl and BDPA radicals have emerged as ideal polarizing agents for these applications due to their narrow line in Electron Paramagnetic Resonance (EPR) spectra, considerable molecular size, and prolonged electron relaxation times.^33^ Recent theoretical studies of the solid effect in liquids have emphasized the critical roles of electron and nuclear spin relaxation times, as well as molecular diffusion, in influencing the shaping and intensity of the DNP field profile.^34–36^

In this study, we explore three distinct biological systems: ATP, a 25-mer DNA duplex, and DMPC lipid bilayers (see Figure 1) by employing solid-effect DNP on phosphorus-31 in its phosphate form. The target molecules vary in terms of molecular size, the number of nonequivalent ^31^P nuclei present, and the mobility of these nuclei. We selected triarylmethyl radicals, specifically Finland and OX063, as polarizing agents based on several compelling criteria: (i) they provide high solid-effect DNP enhancements, as previously demonstrated in glycerol solutions,^31^ (ii) they exhibit low toxicity in biological systems, with OX063 being particularly advantageous in this respect,^37^ and (iii) they are highly stable in aqueous solution.^38^ These combined properties make triarylmethyl radicals the optimal choice for conducting DNP studies of biological molecules under near-physiological conditions.

The liquid-state DNP experiments were performed using a home-built DNP spectrometer operating at a magnetic field strength of 9.4 T,^39,40^ corresponding to a ^31^P Larmor frequency of 162 MHz. Key features of this spectrometer include a Fabry–Pérot microwave resonator, an ultrathin sample layer (20 μm, with a total sample volume of ∼200 nL), and extensive nitrogen gas cooling.^41^ These elements collectively enable effective temperature stabilization at around 42 °C throughout the entire measurement time, which lasted up to 5 h, while applying 5 W of continuous-wave microwave irradiation at 263 GHz. The magnetic field position for the direct solid effect was precisely optimized by utilizing glycerophosphate in aqueous solutions and adjusted to align with the positive ^31^P NMR signal, specifically at 9.422 T (see Figure 2). Notably, the Overhauser effect was not detected at the midpoint of the field profile.

**Figure 2:**
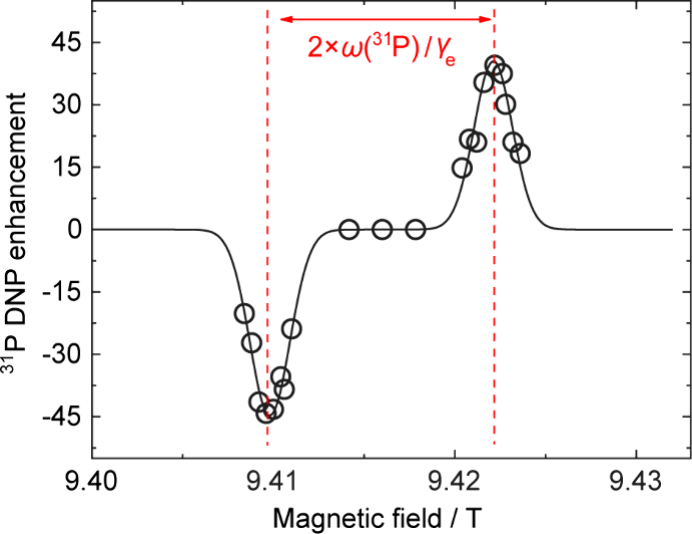
Field profile of the ^31^P DNP enhancement observed in a 6 M glycerophosphate aqueous solution, doped with 100 mM OX063 radicals. The measurements were conducted at a magnetic field strength of 9.4 T and a temperature of approximately 42 °C. The antisymmetric peaks in the field profile are spaced by 2 × ω_P_/γ_e_, indicating that the DNP mechanism is the solid effect. The enhancement for the ^31^P signal reached a maximum of ±45.

Figure 3 presents the hyperpolarized ^31^P NMR spectra obtained for ATP and 25-mer DNA in aqueous solutions at a magnetic field strength of 9.4 T. The experiments involved a variation of the concentrations of ATP (1 M) and 25-mer DNA (5 mM) samples, while the concentration of OX063 radical was set to 100 mM and calibrated using continuous-wave X-band EPR. Despite experiencing NMR line broadening due to magnetic field inhomogeneity, we successfully resolved distinct ^31^P lines in the ATP spectrum, indicating the presence of nonequivalent phosphorus nuclei: alpha, beta, and gamma (see Figure 1B). Interestingly, we observed comparable ^31^P DNP enhancements (∼20) for both ATP and DNA samples, which was unexpected given that larger molecules typically exhibit greater solid-effect DNP efficiency.^31^ This finding can be largely attributed to differences in sample viscosity, which significantly impact molecular diffusion and, consequently, the effectiveness of polarization transfer. A viscosity analysis using EPR spectroscopy revealed that the 1 M ATP sample exhibited a viscosity of approximately 6 cP, while the 5 mM DNA sample had a lower viscosity of about 1 cP (see details in the Supporting Information). Upon examining the ATP sample at a reduced concentration of 0.2 M, which corresponded to the viscosity of the DNA sample, we observed a substantial decrease in the ^31^P DNP enhancement, with a value of approximately 4 (see Figure S3 in the SI). These findings underscore the delicate interplay between analyte molecular size and viscosity in achieving optimal DNP enhancements. While larger molecules benefit from slower diffusion rates, which enable the effective solid effect to occur even in low-viscosity media (approximately 1 cP), smaller molecules like ATP require a minimum viscosity of around 6 cP to ensure efficient hyperpolarization transfer. These promising results provide a strong foundation for further exploration of liquid-state DNP techniques within the field of biomolecular NMR spectroscopy.

**Figure 3:**
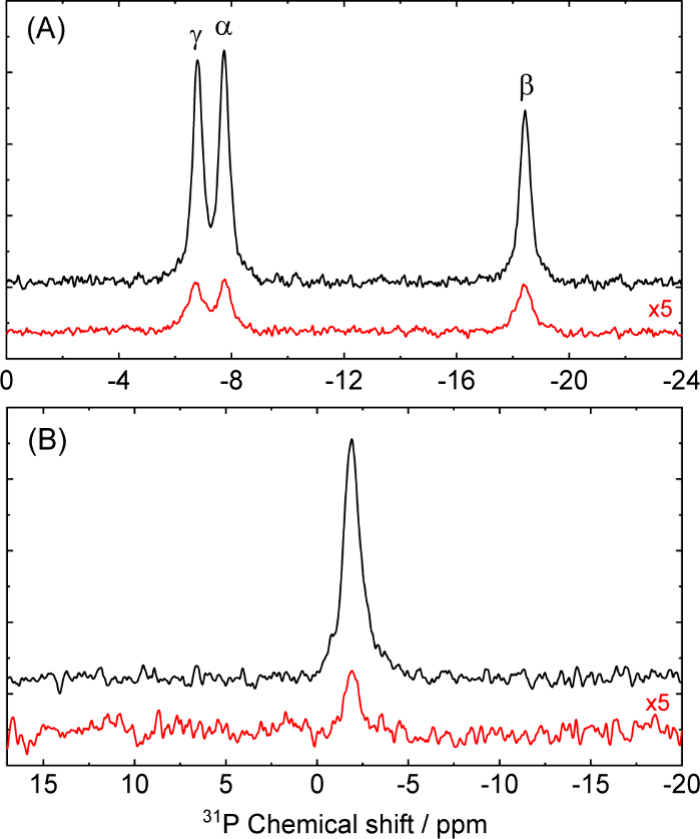
^31^P NMR spectra recorded at a magnetic field of 9.4 T for (A) adenosine triphosphate (1 M) and (B) 25-mer DNA duplex (5 mM), each codissolved with the OX063 radical (100 mM) in deuterated water at a sample temperature of 42 °C. The spectra are shown with microwave on (black) and off (red). The microwave power was set to 5 W at a frequency of 263 GHz. The NMR signals are normalized with respect to the number of acquisitions (with microwave ∼10,000, without microwave ∼80,000).

Additionally, the DNP experiments conducted here provide valuable insights into the potential application of the solid effect on ^31^P nuclei within heterogeneous systems, thereby extending our research focus beyond homogeneous solutions. We investigated a system consisting of DMPC lipid bilayers and Finland radical in aqueous solutions with a molar ratio of 1:20 for radical to phospholipid. The increased line width observed in the ^31^P NMR spectrum is attributed to dipolar coupling and chemical shift anisotropy arising from the restricted molecular flexibility of the lipid headgroup. The solid-effect ^31^P DNP experiments performed with a DMPC/Finland sample in the fluid phase showed promising results, allowing us to detect an anisotropic line (see Figure 4) even with a sample volume limited to approximately 200 nanoliters. Importantly, the absence of a detectable signal in a reference Boltzmann NMR spectrum for the same sample underscores the advantage of DNP techniques in identifying weak NMR signals. This finding suggests that liquid-state DNP could be a powerful tool in scenarios where conventional NMR lacks sufficient sensitivity, particularly in systems with limited sample volumes.

**Figure 4:**
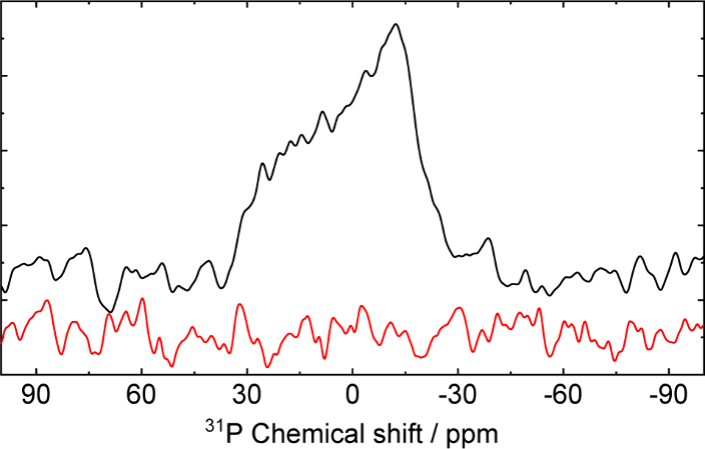
^31^P NMR spectra recorded at a magnetic field of 9.4 T for DMPC lipid bilayers doped with Finland radical in deuterated water with a molar ratio of 1:20 for radical:lipid at a sample temperature of 42 °C. The spectra are shown with microwave on (black) and off (red). The microwave power was set to 5 W at a frequency of 263 GHz. A total of ∼16,000 acquisitions were performed for both NMR spectra.

In this paper, we successfully demonstrated the feasibility of using DNP to investigate biomolecules under near-physiological conditions at a nanoliter scale and high magnetic fields. This advancement is particularly significant given the NMR challenges of working with low-γ nuclei and small sample volumes. One of the most remarkable aspects of our results is the ability to detect as little as ∼1 nanomole of target molecules, highlighting the effectiveness of the developed DNP approach. Furthermore, we anticipate that future research involving higher molecular weight analytes will allow for even greater reductions in the required sample amounts. The considerable potential of solid-effect DNP promises to expand its applications, particularly in elucidating the behavior and dynamics of biomolecules, as well as binding interactions between small molecules, such as ATP or short oligonucleotides, and macromolecules like proteins or native membranes.

In conclusion, our research presents a significant advancement in liquid-state NMR by achieving the first hyperpolarization of ^31^P nuclei in phosphate groups through the solid-effect DNP at high magnetic fields. We have highlighted three key findings: (i) the successful investigation of nonviscous aqueous systems at room temperature, (ii) the hyperpolarization of phosphorus-31 in its pentavalent form, and (iii) the study of biologically relevant molecules. These results pave the way for new applications of solid-effect DNP beyond traditional solid-state environments, potentially enabling nanoliter-scale NMR techniques at high magnetic fields. Looking ahead, it is essential to further investigate and optimize the parameters that influence the efficiency of solid-effect DNP across various molecular environments, both homogeneous and heterogeneous. This will allow us to fully grasp the potential of this new approach and gain deeper insight into complex biological systems.

## Supplementary Material



## Notes

More details on the sample preparation, as well as the EPR, NMR, and DNP experimental procedures, along with additional EPR and DNP data (PDF)

## Abbreviations

NMR: nuclear magnetic resonance
DNP: dynamic nuclear polarization
EPR: electron paramagnetic resonance
BDPA: α,γ-bisdiphenylene-β-phenylallyl
ATP: adenosine triphosphate
DNA: deoxyribonucleic acid, DMPC, 1,2-dimyristoyl-*sn*-glycero-3-phosphocholine
