# Supplementary material for: 31P Dynamic Nuclear Polarization through the Solid Effect: Study of Biomolecules in Aqueous Solutions at 9.4 T
Source: Anal Chem. 2025 Jul 9;97(28):14890–3. doi: 10.1021/acs.analchem.5c02158 (PMC12291044; doi:10.1021/acs.analchem.5c02158)
Supplement: Supplementary file 1 [file ac5c02158_si_001.pdf]

^{31}P Dynamic Nuclear Polarization through the Solid Effect: Study of Biomolecules in Aqueous Solutions at 9.4 T

Andrei Kuzhelev*

Goethe University Frankfurt am Main, Institute of Physical and Theoretical Chemistry, Center for Biomolecular Magnetic Resonance, Max von Laue Str. 7, 60438 Frankfurt am Main, Germany.
kuzhelev@chemie.uni-frankfurt.de

Table of contents

Experimental Section S2

Sample Preparation S2

Instrumental Setup S4

Sample Temperature Calibration S5

^{31}P NMR of 0.2 M ATP S7

Analysis of Viscosity S8

References S11

Experimental Section

Sample Preparation

Materials

Triarylmethyl radicals, specifically the Finland and OX063 radicals, were obtained from Polarize. Adenosine triphosphate (ATP) in the form of disodium salt trihydrate, β-glycerophosphate disodium salt hydrate, 3-carboxy-proxyl, and glycerol were sourced from Sigma-Aldrich. The 25-mer oligonucleotide sequences were purchased from Eurogentec, and 1,2-dimyristoyl-sn-glycero-3-phosphocholine (DMPC) was acquired from Avanti Polar Lipids.

Polarizing Agents

The Finland radical was employed as a polarizing agent for lipid sample, while the OX063 radical was used for glycerophosphate, ATP and DNA samples to prevent aggregation issues that arise with Finland radicals in those environments. The concentration of OX063 in these samples was calibrated using continuous-wave X-band EPR spectroscopy at room temperature.

Glycerophosphate Preparation

A solution of glycerophosphate was prepared at concentration of 6 M in deuterated water. Solid OX063 radical was then added to this solution, achieving a concentration of 100 mM.

Adenosine Triphosphate (ATP) Preparation

Solutions of ATP were prepared at concentrations of 1 M and 0.2 M in deuterated water. Solid OX063 radical was then added to these solutions, achieving a concentration of 100 mM.

25-mer DNA Preparation

The oligonucleotide base sequences were carefully optimized to ensure that the melting temperature (T_m) of the double strands exceeded the DNP experimental temperature, with the 25-mer DNA exhibiting a T_m of 59.1 °C. Double-stranded DNA was formed from complementary single-stranded oligonucleotides using a standard annealing protocol: the solution was heated to 95 °C for 5 minutes and then gradually cooled to room temperature over a duration of <60 minutes. The annealing buffer consisted of 100 mM NaCl and 10 mM phosphate buffer to promote optimal hybridization into stable double-stranded DNA. Following the annealing process, excess water was removed using a SpeedVac vacuum concentrator. A final concentration of 5 mM DNA was achieved by rehydrating the dried oligonucleotides with

a deuterated phosphate buffer containing sodium chloride. Solid OX063 radical was then added to the rehydrated solution, ensuring a concentration of 100 mM.

Viscosity analysis

Solutions of 0.5 mM 3-carboxy-proxyl were prepared in water/glycerol mixtures with glycerol concentrations of 0%, 10%, 20%, 30%, 40%, 45%, and 50%. The glycerol was accurately weighed using a scale. Additionally, ATP and DNA samples were prepared in the presence of 3-carboxy-proxyl radical. For this, ATP was initially dissolved in deuterated water at concentrations of 1 M and 0.2 M, while DNA was dissolved at a concentration of 5 mM. Solid 3-carboxy-proxyl radical was then added to these solutions to achieve a final concentration of 0.5 mM.

Lipid Bilayer Preparation

To prepare lipid bilayers, a mixture of 5.5 mg of dry DMPC phospholipid powder and 0.4 mg of Finland radicals was dissolved in 100 µl of chloroform and 100 µl of methanol. The solution was vortexed for 3-5 minutes until a macroscopically homogeneous liquid phase was obtained. The solvents were then evaporated under a stream of nitrogen gas, resulting in the formation of a thin lipid film at the bottom of the flask. To remove any residual solvent, the flask was evacuated for a minimum of 3 hours at a pressure of 5×10^{-6} bar. The dry DMPC/Finland lipid film was subsequently hydrated with 7.3 mg of D_2O and allowed to rest at room temperature for one hour, facilitating liposome formation in the presence of atmospheric oxygen. The gel-to-liquid phase transition temperature of the DMPC lipid bilayers is approximately 297 K, ensuring that the samples remain in the fluid phase under DNP conditions.

Instrumental Setup

EPR Experiments

Continuous-wave X-band (0.33 T) EPR experiments were performed at room temperature using a Bruker Elexsys E500 spectrometer. The experimental parameters were carefully optimized as follows: frequency set to 9.331 GHz, sweep width of 10 mT, microwave power at 2 mW, modulation frequency of 100 kHz, modulation amplitude of 0.05 mT, time constant of 40.96 ms, conversion time of 40.96 ms, sweep time of 41.94 s, with a total of 1024 data points and one scan per measurement. Analysis of the EPR line broadening was conducted using the Easyspin software package.[1]

DNP/NMR Experiments

DNP-enhanced NMR spectra were recorded on a custom-built DNP spectrometer, based on a Bruker Avance II wide-bore spectrometer operating at a magnetic field strength of 9.4 T.[2] Microwaves for the DNP experiments were generated using a gyrotron (Gycom) operating at a frequency of 263 GHz and power of 5 W.[3] The DNP probehead was a home-built Fabry-Perot/stripline triple resonance structure, designed to operate at electron frequency of 260 GHz, proton frequency of 400 MHz, and carbon-13 or phosphorus-31 frequencies of 100/162 MHz, respectively.[4] The stripline with a high heat conductivity was additionally cooled by a flow of room temperature nitrogen gas.

For each measurement, NMR free induction decay (FID) signals were recorded under two conditions: with and without microwave irradiation. Excitation was achieved using a standard 90° radiofrequency pulse, with a pulse length of 90 μs and a repetition time was optimized for each NMR/DNP experiments and set to 1 second. The DNP enhancement was quantitatively assessed by integrating the area under all phosphorus-31 signals. The enhancement factor was calculated using the formula $\varepsilon = (I/I_0) - 1$, where I represents the integrated area of the signal with microwave irradiation, and I_0 represents the integrated area without microwave irradiation.

The number of scans varied according to the sample type. For ATP and DNA samples, approximately 10,000/80,000 scans were recorded for ^{31}P DNP/NMR, corresponding to measurement time of around 3 hours / 24 hours, respectively. For lipid samples, around 16,000 scans were conducted in both ^{31}P DNP/NMR experiments, resulting in approximately 5 hours of measurement time.

Sample Temperature Calibration

To calibrate the sample temperature during continuous-wave microwave irradiation at a frequency of 263 GHz and power of 5 W, a 3:7 mixture of glycerol and D_2O, doped with 24 mM OX063, was utilized. The calibration curve was established by monitoring the temperature increase through the differential chemical shift between the CH/CH_2 peak and the OH peak of glycerol (see Figure S1). For this purpose, ^1H NMR spectra were acquired in the Fabry-Pérot/stripline probehead while systematically varying the sample temperature from 27 °C to 57 °C using hot nitrogen gas (without microwave irradiation).

DNP-enhanced NMR measurement was conducted on the same glycerol/D_2O mixture (with microwave irradiation). During this experiment, the differential chemical shift was measured, yielding a value of $\Delta\delta = 435 \pm 5$ Hz (with fixed room temperature nitrogen gas, see Figure S2). By applying the linear fit parameters derived from the calibration curve (refer to Figure S1), it was inferred that the actual sample temperature during the DNP measurement was approximately 42 ± 2 °C.

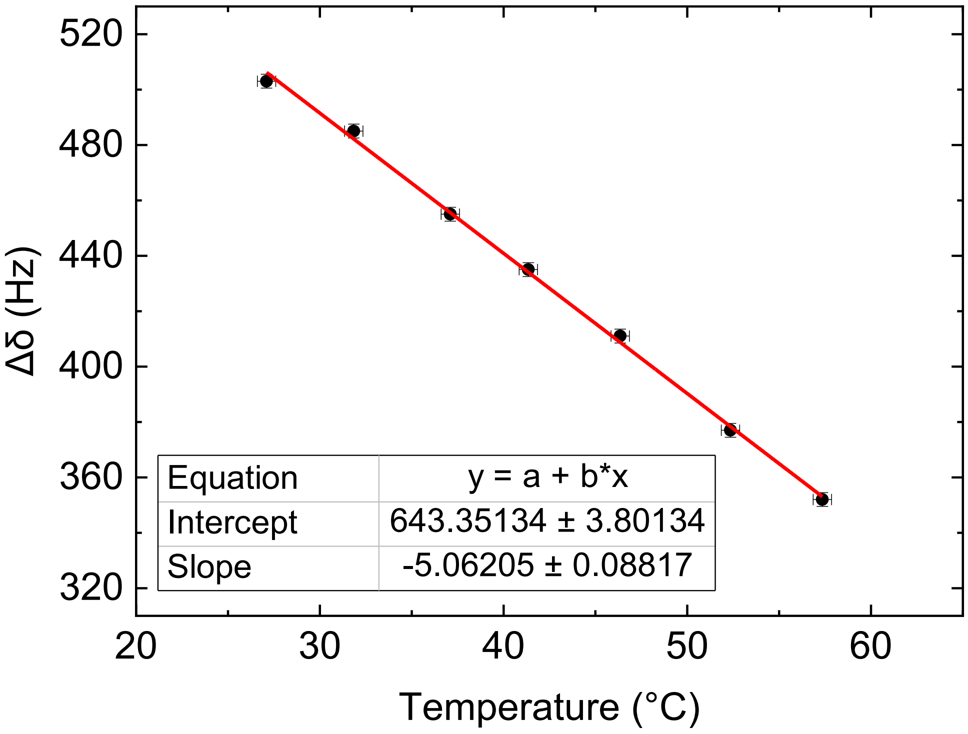

Figure S1. Calibration of sample temperature utilizing a 3:7 mixture of glycerol and D_2O, doped with 24 mM OX063 radical. The differential chemical shift between the CH/CH_2 peak and the OH peak of glycerol was plotted as a function of temperature and fitted with a linear regression.

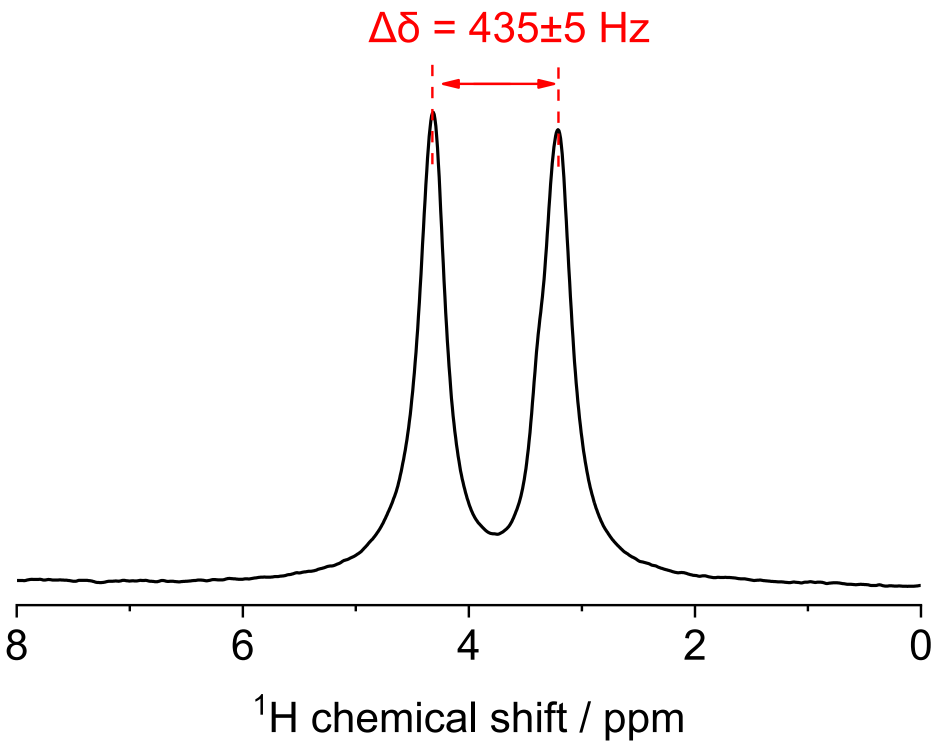

Figure S2. ^{1}H DNP spectrum of a 3:7 mixture of glycerol and D_2O, doped with 24 mM OX063 radical. The experiment was performed under microwave irradiation at 263 GHz and a power of 5 W, using room temperature nitrogen gas for cooling. The sample temperature was estimated to be 42 ± 2 °C, based on the intercept and slope obtained from Figure S1.

^{31}P NMR of 0.2 M ATP

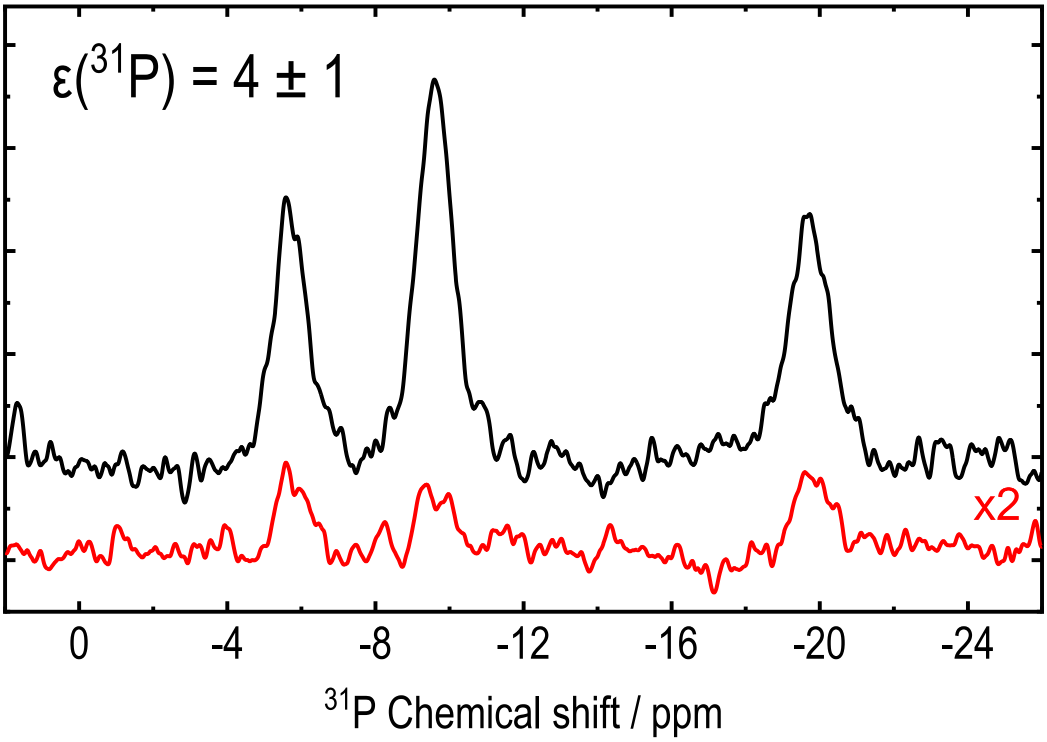

Figure S3. ^{31}P NMR spectra recorded at a magnetic field of 9.4 T for adenosine triphosphate (0.2 M) co-dissolved with the OX063 radical (0.1 M) in aqueous solution at a sample temperature of 42 °C. The spectra are shown with microwave on (black) and off (red). The microwave power was set to 5 W at a frequency of 263 GHz. The NMR signals are normalized with respect to the number of acquisitions (with microwave ~20,000, without microwave ~150,000).

Analysis of Viscosity

The analysis of EPR spectra of nitroxides reveals significant variations in linewidths and lineshapes as a function of the rotational correlation time, τ_R. In low viscosity solutions, nitroxides experience rapid tumbling, resulting in nearly complete averaging of g- and hyperfine anisotropies. However, as viscosity increases, the tumbling slows down, leading to less effective averaging. This results in unequal widths of the three nitroxide lines,[5] reflecting changes in the environment's viscosity.

To establish a correlation between viscosity and tumbling correlation time of nitroxide, we conducted EPR experiments at room temperature using 3-carboxy-proxyl and water/glycerol mixtures. The lineshape analysis was performed using Easyspin software,[1] and a linear fit was plotted to represent the relationship between correlation time and viscosity (see Figure S4). Viscosity values for the water/glycerol mixtures were obtained from previous studies.[6]

Further investigations included X-band EPR measurements of ATP and DNA samples, both doped with 0.5 mM 3-carboxy-proxyl, under identical experimental conditions (see Figure S5-6). The rotational correlation time of the nitroxide was quantified, yielding values of ~100 ps for 1 M ATP and ~28 ps for both 0.2 M ATP and 5 mM DNA. Utilizing the parameters derived from the calibration curve (see Figure S4), we concluded that the viscosity of the 1 M ATP sample in aqueous solution was approximately 6 cP, while both 0.2 M ATP and 5 mM DNA samples exhibited a viscosity of around 1 cP.

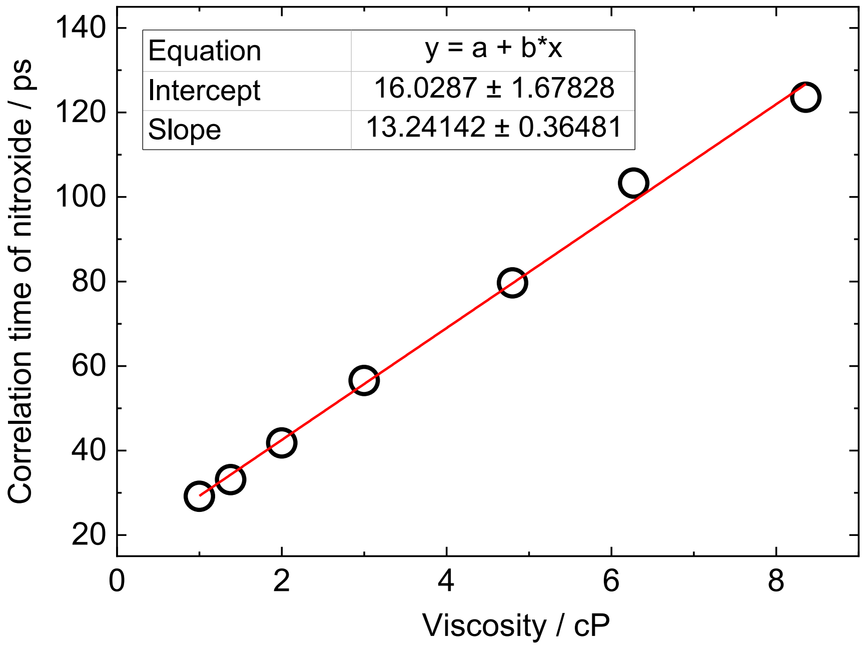

Figure S4. Calibration of sample viscosity utilizing mixtures of glycerol and water, doped with 0.5 mM 3-carboxy-proxyl. The rotational correlation times derived from the simulation of the corresponding EPR spectra were plotted as a function of viscosity. The measurements were performed using X-band EPR at room temperature.

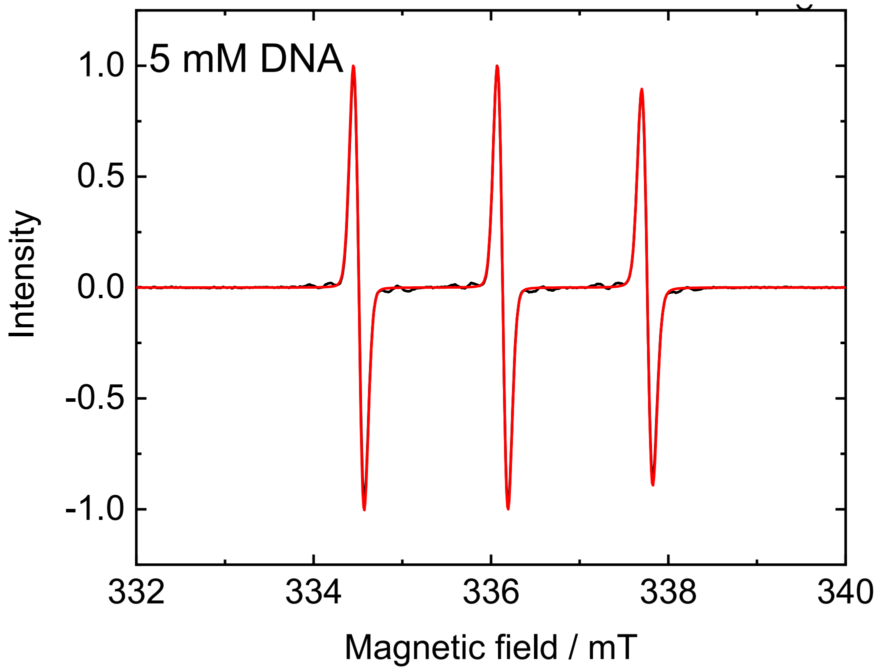

Figure S5. The X-band continuous wave EPR spectrum of 0.5 mM 3-carboxy-proxyl in an aqueous solution of 5 mM DNA, with the experimental data shown as black lines and the corresponding simulation depicted in red. The analysis of the spectrum revealed a rotational correlation time for the nitroxide of 28 ps.

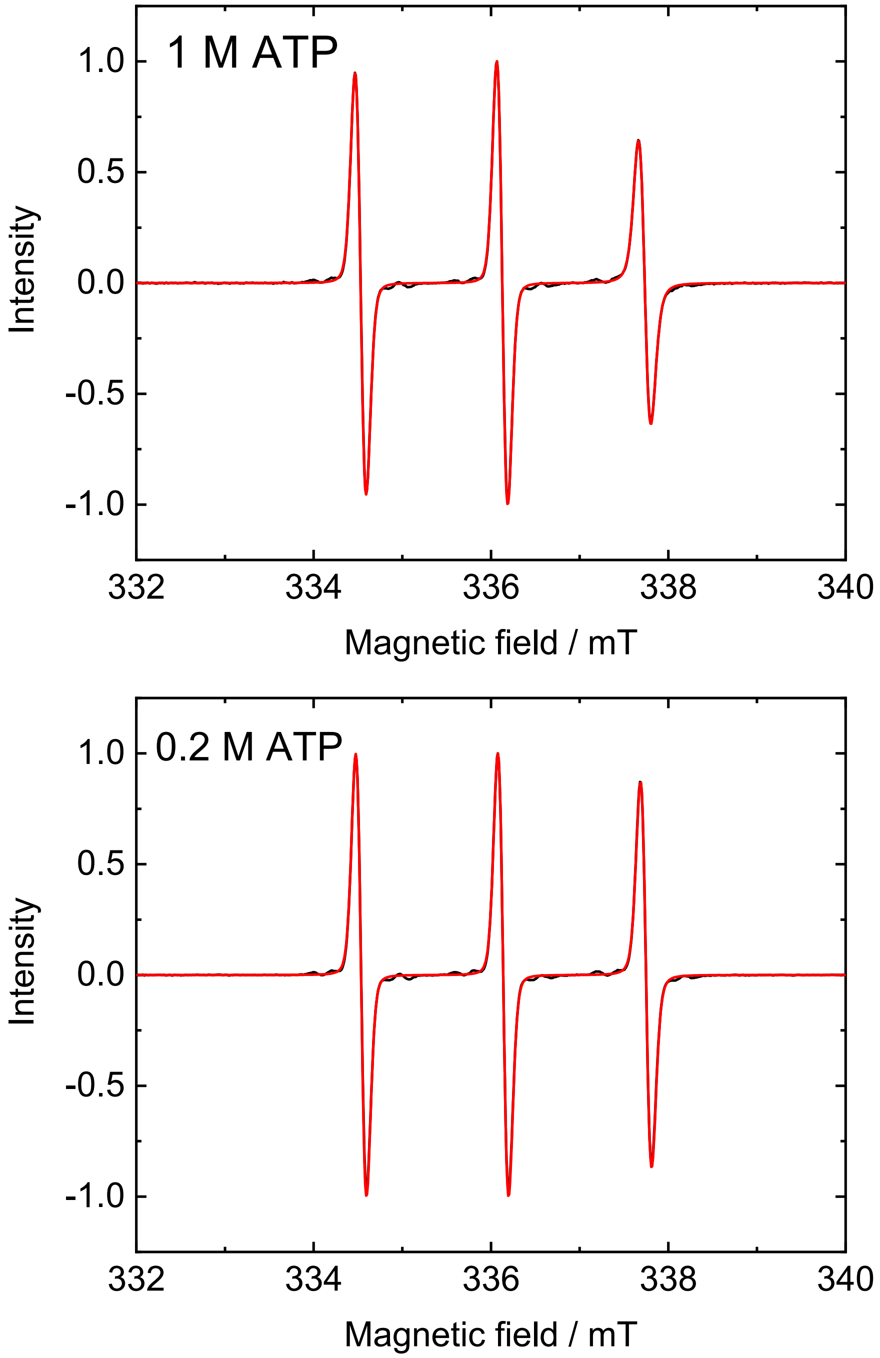

Figure S6. X-band continuous wave EPR spectra of 0.5 mM 3-carboxy-proxyl in aqueous solutions of 1 M and 0.2 M ATP, with the experimental data shown as black lines and their corresponding simulations depicted in red. The analysis of these spectra revealed rotational correlation times for the nitroxide of 100 ps for the 1 M ATP sample and 28 ps for the 0.2 M ATP sample, respectively.

References

(1) Stoll, S.; Schweiger, A. EasySpin, a Comprehensive Software Package for Spectral Simulation and Analysis in EPR. *J. Magn. Reson.* **2006**, *178* (1), 42–55.

(2) Denysenkov, V. P.; Prandolini, M. J.; Krahn, A.; Gafurov, M.; Endeward, B.; Prisner, T. F. High-Field DNP Spectrometer for Liquids. *Appl. Magn. Reson.* **2008**, *34* (3–4), 289–299.

(3) Denysenkov, V.; Prandolini, M. J.; Gafurov, M.; Sezer, D.; Endeward, B.; Prisner, T. F. Liquid State DNP Using a 260 GHz High Power Gyrotron. *Phys. Chem. Chem. Phys.* **2010**, *12* (22), 5786–5790.

(4) Denysenkov, V.; Dai, D.; Prisner, T. F. A Triple Resonance (e, 1H, 13C) Probehead for Liquid-State DNP Experiments at 9.4 Tesla. *J. Magn. Reson.* **2022**, *337*, 107185.

(5) Schneider, D. J.; Freed, J. H. Calculating Slow Motional Magnetic Resonance Spectra. In *Spin Labeling: Theory and Applications*; Springer, 1989; pp 1–76.

(6) Cheng, N. S. Formula for the Viscosity of a Glycerol-Water Mixture. *Ind. Eng. Chem. Res.* **2008**, *47* (9), 3285–3288.
